# Charge-Balanced Electrical Stimulation Can Modulate Neural Precursor Cell Migration in the Presence of Endogenous Electric Fields in Mouse Brains

**DOI:** 10.1523/ENEURO.0382-19.2019

**Published:** 2019-12-13

**Authors:** Stephanie N. Iwasa, Abdolazim Rashidi, Elana Sefton, Nancy X. Liu, Milos R. Popovic, Cindi M. Morshead

**Affiliations:** 1 Institute of Biomaterials and Biomedical Engineering, University of Toronto, Toronto, Ontario M5S 3G9, Canada; 2 KITE, Toronto Rehabilitation Institute, University Health Network, Toronto, Ontario M5G 2A2, Canada; 3 Department of Surgery, University of Toronto, Toronto, Ontario M5T 1P5, Canada; 4 Donnelly Centre for Cellular and Biomolecular Research, University of Toronto, Toronto, Ontario M5S 3E1, Canada; 5 CRANIA, University Health Network & University of Toronto, Toronto, Ontario M5G 2A2, Canada

**Keywords:** cell migration, corpus callosum, electric fields, electrical stimulation, neural stem cells

## Abstract

Electric fields (EFs) can direct cell migration and are crucial during development and tissue repair. We previously reported neural precursor cells (NPCs) are electrosensitive cells that can undergo rapid and directed migration towards the cathode using charge-balanced electrical stimulation *in vitro*. Here, we investigate the ability of electrical stimulation to direct neural precursor migration in mouse brains *in vivo*. To visualize migration, fluorescent adult murine neural precursors were transplanted onto the corpus callosum of adult male mice and intracortical platinum wire electrodes were implanted medial (cathode) and lateral (anode) to the injection site. We applied a charge-balanced biphasic monopolar stimulation waveform for three sessions per day, for 3 or 6 d. Irrespective of stimulation, the transplanted neural precursors had a propensity to migrate laterally along the corpus callosum, and applied stimulation affected that migration. Further investigation revealed an endogenous EF along the corpus callosum that correlated with the lateral migration, suggesting that the applied EF would need to overcome endogenous cues. There was no difference in transplanted cell differentiation and proliferation, or inflammatory cell numbers near the electrode leads and injection site comparing stimulated and implanted non-stimulated brains. Our results support that endogenous and applied EFs are important considerations for designing cell therapies for tissue repair *in vivo.*

## Significance Statement

The study of electricity in biological environments outside of the well-known action potential is becoming more prominent. Applied electrical stimulation is used clinically and can modulate cell behavior. Endogenous electric fields (EFs) exist in the adult brain along the rostral migratory stream (RMS) and disrupting them can reverse the migration direction of neural precursor cells (NPCs). We demonstrate that an endogenous EF exists on the corpus callosum which correlates with the preferred lateral migration of transplanted NPCs. Endogenous EFs in the brain provide migratory cues that can impact neural repair.

## Introduction

Galvanotaxis is the directed migration of cells in electric fields (EFs). EFs are physiologically relevant and are critical in development and wound healing (McCaig et al., 2005; Iwasa et al., 2017). In models of injury, EFs generated as a result of epithelia damage promote wound closure. Disrupting these EFs prevents wound closure. Despite the presence of injury-related secreted chemotactic factors, cells do not migrate to the site of injury (Zhao, 2009). This demonstrates the necessity and the overriding signaling nature of EFs (Zhao et al., 2006; Zhao, 2009).

In the context of the central nervous system, *in vitro* studies demonstrate that undifferentiated neural precursor cells (NPCs) are electrosensitive cells that migrate rapidly (∼1 μm/min) to the negative pole (cathode) in the presence of an applied direct current EF (Babona-Pilipos et al., 2011, 2018). NPCs are found in the well-defined periventricular region in the adult brain (Morshead et al., 2003). *In vivo,* NPCs migrate along the rostral migratory stream (RMS) to the olfactory bulb where they give rise to interneurons (Lois and Alvarez-buylla, 1994). An endogenous EF exists along the RMS and contributes to NPC migration to the olfactory bulb (Cao et al., 2013). Together, the *in vitro* and *in vivo* data supports the hypothesis that EF application can modify NPC behavior and could contribute to neural repair.

Commonly-used direct current EFs can cause tissue damage and electrode degradation through charge accumulation which can drive electrochemical reactions that can degrade the electrode. Charged-balanced stimulation can reduce the amount of non-reversible reactions at the electrode-tissue interface by balancing the charge in the anodal and cathodal phase (i.e., the amount of charge injected into the tissue is the amount of charge drawn out; Brocker and Grill, 2013; Bertucci et al., 2019). Thus, the use of charge-balanced EFs is an attractive approach to stimulate cells *in vivo*. Indeed, we have demonstrated that NPCs migrate in charge-balanced biphasic monopolar stimulation pulses (Babona-Pilipos et al., 2015) *in vitro*, providing support for more detailed investigation of this wave form for clinical application. The aim of this study is to examine endogenous EFs and to determine whether biphasic charge-balanced electrical stimulation can direct NPC migration in the adult mouse brain. Given previous work that demonstrated the corpus callosum is amenable to the migration of transplanted cells (Modo et al., 2004); here, we examined transplanted fluorescent NPC migration on the corpus callosum in response to applied electrical stimulation.

We found that transplanted NPCs exhibited a marked propensity to survive and migrate laterally along the corpus callosum following transplantation *in vivo*, irrespective of exogenous EF application. We further demonstrate the presence of an endogenous electrical potential difference along the corpus callosum, which correlates with the robust lateral migration we observed following transplantation. We asked if application of an exogenous EF could promote the migration of cells medially, away from their default lateral migratory pathway. Stimulating electrodes were implanted into the cortex above the corpus callosum following cell transplantation and the relative number, location, and differentiation profile of transplanted cells was examined in two stimulation paradigms (Babona-Pilipos et al., 2015). Interestingly, we observed a small but significant increase in cathodally (medially) directed migration of transplanted NPCs with cortical stimulation. Similar to our previous report (Morrison et al., 2019), the charge-balanced biphasic monopolar stimulation did not result in enhanced inflammation as measured by microglia/macrophage number. Overall, our findings reveal cell migration patterns in response to endogenous and applied electrical stimulation, highlighting the potential impact of EFs in modifying cell behavior *in vivo*.

## Materials and Methods

### Overview

**Figure 1.**
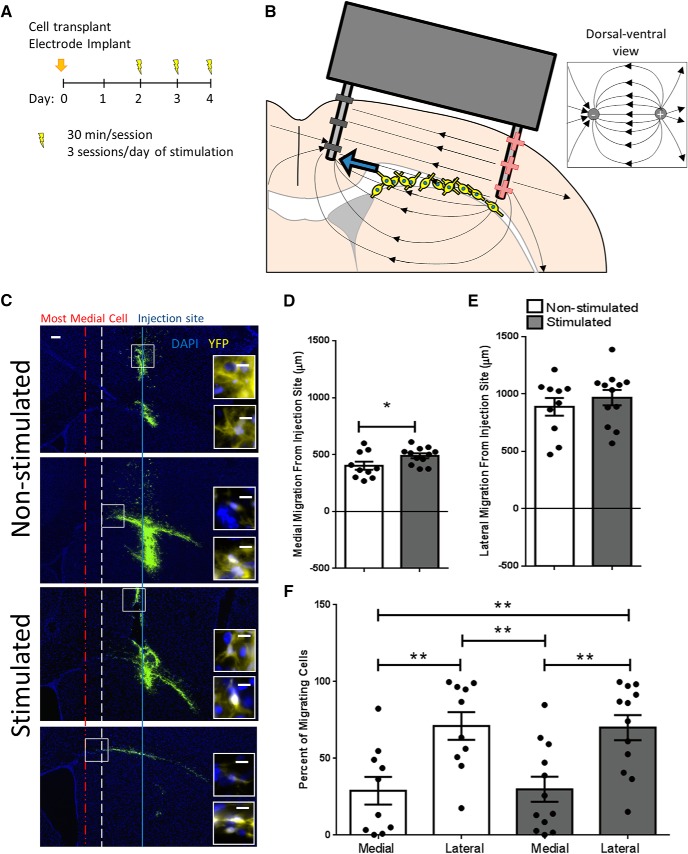
Three-day electrical stimulation paradigm increases medial migration. ***A***, Experimental 3-d stimulation paradigm using electrical stimulation as seen in Extended Data Figure 1-1. ***B***, Schematic of the transplanted cells and electrode are featured on a coronal brain section. The EF lines from two parallel wires in a homogenous medium are superimposed on the brain with thin black arrows. Predicted migration direction due to stimulation is indicated by the thick blue arrow. ***C***, Representative sections of injection site and most medially located cells on the corpus callosum of a non-stimulated and stimulated brain. Solid blue line = injection site, white dashed line = most medial cell in a non-stimulated brain, red dashed and dotted line = most medial cell in a stimulated brain. Scale bar = 200 μm. Inset images of sample cells from the boxed region. Scale bar = 10 μm. Significantly further maximum medial cell migration toward the cathode^a^ (***D***) and no significant difference in maximum lateral cell migration toward the anode^b^ (***E***) of implanted non-stimulated (*n* = 10) and stimulated brains (*n* = 12). Each point in the graph represents the farthest cell in one mouse brain, plotted with mean ± SEM. Unpaired *t* test, equal variance was used (*p = 0.045). ***F***, Percentages of cells on the medial and lateral side of the injection site^d^. There were significantly more cells on the lateral compared to medial side of the injection site. Cells were analyzed in the section where the most medial cell was found in each non-stimulated (*n* = 10) and stimulated (*n* = 12) brain. Each point in the graph represents the percentage of cells on the medial or lateral side of the injection site in a brain, plotted with mean ± SEM. A multiple comparisons one-way ANOVA test with Tukey’s *post hoc* corrections was used (**p < 0.01).

To examine the effects of biphasic monopolar charge-balanced stimulation on directed migration *in vivo*, we first transplanted NPCs derived from transgenic mice expressing yellow fluorescent protein (YFP) onto the corpus callosum of adult wild-type mice with a small deposit of cells also transplanted into the cortex. NPCs have been shown not to migrate in the cortex (Cayre et al., 2006). Therefore, we used the transplanted cells in the cortex as a reference point for the cell injection site and examined the behavior of YFP+ transplanted cells on the corpus callosum. At the time of cell transplantation, electrodes were implanted into the cortex with the cathode and anode leads located 1 mm medial and lateral to the cell injection site, respectively, and stimulated with a predicted EF lines as seen in Figure 1*A–C*. Two days after surgery, brains were electrically stimulated using a biphasic monopolar charge-balanced wave form for 3 d, using a similar wave form to what was used *in vitro* to promote rapid and directed NPC migration (Babona-Pilipos et al., 2015; Extended Data Fig. 1-1). The EF strength was 250 mV/mm at the cathodal peak. Our main aim was to determine whether electrical stimulation could promote NPC migration toward the cathode *in vivo*.

10.1523/ENEURO.0382-19.2019.f1-1 Extended Data Figure 1-1 Measured voltage waveform across the implanted electrode. Biphasic monopolar waveform consisting of a cathodal pulse with four times the amplitude of the anodal pulse. Pulse width of the anodal pulse is four times the duration in order to have a charge-balanced waveform. Download Figure 1-1, TIF file.

Based on previous work (Babona-Pilipos et al., 2011, 2012, 2018), we predicted that electrical stimulation would result in YFP+ cell migration toward the midline (cathode) in electrically stimulated brains compared to implanted non-stimulated control brains. Medial-lateral distances were measured from the interhemispheric midline to (1) the most medial cell in the corpus callosum and (2) to the center of the cortex cell deposit in all sections with YFP+ cells and (3) the most lateral cell in the corpus callosum. The distance between the average injection site and the closest medial cell and farthest lateral cell was calculated for each brain. Following the primary analysis, we examined other behaviors of the transplanted cells and endogenous factors around implant and transplant sites.

### Animals

All animal work was approved by the University of Toronto Animal Care Committee in accordance with institutional guidelines (protocol no. 20011279). The ethical standards governing this reported research at the University of Toronto are in accordance with the federally mandated standards (Canadian Council of Animal Care), provincial legislation (Animals for Research Act, R.S.O. 19990, c.A.22) and the Local Animal Care Committee. NPCs were isolated from dissections of the adult periventricular region of transgenic mice expressing YFP (7AC5/EYFP) bred in house. Surgeries were performed on C57BL/6 male mice aged 7–11 weeks (Charles River). Endogenous electric potential measurements were performed on C57BL/6 mice aged 11–13 weeks.

### Electrode construction

Electrodes were constructed as described previously (Iwasa SN, Rashidi A, Sefton E, Popovic MR, and Morshead CM, unpublished observations). Briefly, electrodes were manufactured in house with platinum wires (diameter 127 μm, lot #571752, 767000, A-M Systems) mounted on a 2-mm connector (3M9397-ND, Digikey) using solder (SN60PB40, 0.5 mm, Kester) and soldering paste (lead-free solder paste #5, #48420, NSF-61, 48 g, Oatey). Epoxy glue (Devcon 5 min Epoxy Gel, 14240 25 ml Dev-Tube, Devcon) was used for insulation and support. The cathode and the anode wire were 2.0 ± 0.1 mm apart and 2.0 ± 0.1 mm long, and uninsulated. Measurement electrodes were constructed in a similar manner with a final step of insulating the platinum lead wires with epoxy (Loctite EA E-60NC, Loctite). Four different measuring electrodes were constructed, and each measurement was done with either a different electrode or with freshly cut lead wires.

### Cell culture

NPCs were isolated from periventricular dissection of the adult YFP+ mouse as previously described (Morshead et al., 2002; Babona-Pilipos et al., 2011, 2012). Briefly, the isolated tissue was enzymatically dissociated in hyaluronidase (1157 units/ml, Millipore-Sigma), trypsin (1.33 mg/ml, Millipore-Sigma) and kynurenic acid (0.13 mg/ml, Millipore-Sigma) for 25 min and mechanically dissociated through trituration. The solution was spun down and resuspended in trypsin inhibitor (0.33 mg/ml, Worthington Biochemical Corporation). The suspension was washed with serum-free media (SFM; 1× DMEM/F12, 0.6% glucose, 0.1% NaHCO_3_, 5 mM HEPES buffer, glutamine, defined hormone and salt mixture, and penicillin/streptavidin). The cells were plated in SFM with epidermal growth factor (20 ng/ml; Millipore-Sigma), basic fibroblast growth factor (10 ng/ml; Millipore-Sigma), and heparin (2 μg/ml, Millipore-Sigma) in T25 flasks. The neural precursor colonies (neurospheres) were collected, mechanically dissociated and replated in growth media (passaged) every 6–7 d.

### Cell preparation for transplantation

Neurospheres from passages 1–4 were used for cell transplantation. Approximately 1 h before the start of the surgeries, neurospheres were spun down (1500 RPM, 5 min, room temperature), the supernatant was removed and cells were resuspended in 1-ml Accutase (StemCell Technologies) and incubated for 2 min at 37°C. The tube was removed and flicked for pellet resuspension. The 2-min incubation and pellet resuspension were repeated twice. Cells were resuspended in 2 ml of artificial CSF (aCSF; 124 mM NaCl, 5 mM KCl, 1.3 mM MgCl_2_, 26 mM NaHCO_3_, 10 mM glucose, 2 mM CaCl_2_, and 1% penicillin/streptavidin) and mixed slowly (triturated 10×) with a P1000 pipette to achieve a single cell suspension. Cells were centrifuged (1500 RPM, 5 min, room temperature) and supernatant was removed. Cells were resuspended in 0.250–1 ml of aCSF, counted and spun down (1500 RPM, 5 min, 4°C). The supernatant was removed and replaced with aCSF to achieve a final cell density of 200,000 cells/μl, and cells were placed on ice.

### Surgery for electrode and cell implantation

Surgeries were performed on C57BL/6 mice (Charles River; 22–30 g, 7–11 weeks). Mice were anaesthetized with 5% isofluorane (inhalation). Animals were placed in a stereotactic apparatus under 1.5–2.5% isofluorane (inhalation), given ketoprofen (5.0 mg/kg, subcutaneous), and monitored during surgery. An incision was made along the scalp’s midline. The skull surface was dried using a cotton swab. Using a dental drill (#60 or #77, 1.016 or 0.4572 mm, 8160 or 8177, David Kopf Instruments), three holes were drilled (anterior +0.8 mm, lateral –0.7 mm, –1.7 and –2.7 mm, relative to bregma). The two outer holes were for the electrode leads (lateral –0.7 and –2.7 mm), and the middle hole was for cell transplantation (lateral –1.7 mm).

Before transplantation, the cells were gently stirred or pipetted. A 5.0- or 10.0-μl Hamilton syringe (7762-04, 31 gauge, Hamilton Company) was used to inject 1.2 μl of the YFP+ NPCs onto (1) the corpus callosum (1.0 μl, 2.1 mm deep from the brain’s surface) and (2) into the cortex (0.2 μl, 1.1 mm from the brain’s surface) as the needle was removed. Cells were injected at a rate of 0.2 μl/2 min. Cells were allowed to settle for 10 min on the corpus callosum before the needle was raised to inject 0.2 μl of cells into the cortex which served to mark the injection site, before removal.

Electrode leads were inserted into the outer two drill holes. The insertion of the electrodes was accomplished with reverse action forceps attached to the stereotactic apparatus. The electrodes were lowered into the brain with small turns of the stereotactic. Bone glue (Loctite 454, Alzet; cure time <20 min) or Insta-cure+ cyanoacrylate glue (BSI-106, 14.2 g, Bob Smith Industries; cure time <5 min) was applied to secure the electrode. Once the electrodes were in place, the scalp was sutured closed with 4–0 sterile silk suture and adjacent to the electrode the incision was closed with vetbond tissue adhesive (3M) or Insta-cure+ cyanoacrylate glue.

Following these procedures, mice were housed individually in clean cages and placed under a heating lamp to recover. The mice were monitored until they were awake. They received ketoprofen (5.0 mg/kg, subcutaneously) for the first 12–24 h after surgery for pain relief. Housing enrichments were removed from the cages to reduce agitation of the electrodes. Extra nesting material was placed in cages.

### Electrical stimulation

Beginning 2 d after electrode implantation and cell transplantation, mice received electrical stimulation based on the parameters described by Iwasa and colleagues (Iwasa SN, Rashidi A, Sefton E, Popovic MR, and Morshead CM, our unpublished observations). The implanted electrode was connected to a biphasic electrical stimulator for the duration of stimulation, and mice were anaesthetized with 1.5–2.5% isofluorane during stimulation. Stimulation pulse parameters were under 200-μA amplitude with a ∼500-mV cathodal pulse (500-μs pulse width) and a ∼125-mV anodal pulse (2000-μs pulse width) followed by a ∼1000-ms resting phase similar to our previous *in vitro* report (Babona-Pilipos et al., 2015; Extended Data Fig. 1-1). Stimulation was provided daily, three sessions per day, 30 min per session for 3 or 6 d. Following each stimulation session, mice were returned to their cages, singly housed. Mice were sacrificed within 1–2 h of the last stimulation session by killing via Avertin (250 mg/kg, i.p.) as previously used in animal models of stroke (Nusrat et al., 2018; Vonderwalde et al., 2019). After toe-pinch reflex was lost they were transcardially perfused with 4% paraformaldehyde. Brains were removed and placed in 4% paraformaldehyde for 4 h before transferring to 20% sucrose until sectioning. In all experiments for controls to elucidate the differences created by stimulation alone, non-stimulated implanted mice were treated exactly as described above without electrical stimulation.

### Immunohistochemistry

Sections were stained with primary antibodies: Iba1, rabbit Ab, 1:500 (Wako catalog #019-19741, RRID:AB_839504); GFAP, mouse Ab, 1:1000 (Sigma-Aldrich catalog #G3893, RRID:AB_477010); CC1, mouse Ab, 1:50 (Millipore catalog #OP80, RRID:AB_2057371); GFP, chicken Ab, 1:500 (Aves Labs catalog #GFP-1020, RRID:AB_10000240); DCX, mouse Ab, 1:500 (Santa Cruz Biotechnology catalog #sc-271390, RRID:AB_10610966); and Ki67, rabbit Ab, 1:500 (Abcam catalog #ab16667, RRID:AB_302459). Nuclear staining and mounting were performed with 1:1000 Hoescht (33342, Invitrogen) and Mowiol (Millipore-Sigma) or DAPI (H-1200-10, Vector Laboratories). Negative controls were performed for each antibody with the same procedures without the primary antibodies.

Sections were thawed at room temperature for at least 10 min then rehydrated with PBS. For CC1, Ki67, DCX, and GFP antigen retrieval was performed by placing slides in citrate buffer in a pressure cooker (Nesco Professional) set for 15 min then washed three times, each 5-min wash with PBS and Tween 20 (PBST; D’Amico et al., 2009; Hussaini et al., 2013). Since YFP is a variant of GFP, a GFP antibody was used to label YFP+ cells (Dadwal et al., 2015; Faiz et al., 2015). An antibody was required to visualize the transplanted cells after antigen retrieval was performed. For all antibodies, sections were permeabilized, before blocking, for 20 min in Triton X-100 0.3% (T9284, Millipore-Sigma) or during the block step. Sections were blocked for 1 h with either 5% bovine serum albumin (A9647, Millipore-Sigma) with 0.3 M glycine (GLN001.1, Bioshop) or 5% normal goat serum. Sections were incubated with the primary antibody cocktail overnight at 4°C. The following day, three 5-min PBS or PBST washes were performed and a cocktail of secondary antibodies including combinations of 1:400 goat anti-chicken 488 (Invitrogen catalog #A-11039, RRID:AB_142924), 1:400 goat anti-mouse 568 (Thermo Fisher Scientific catalog #A-11004, RRID:AB_2534072), 1:400 goat anti-rabbit 647 (Thermo Fisher Scientific catalog #A-21245, RRID:AB_2535813) or 1:400 goat anti-rabbit 568 (Thermo Fisher Scientific catalog #A-11036, RRID:AB_10563566), 1:400 goat anti-mouse 647 (Thermo Fisher Scientific catalog #A-21236, RRID:AB_2535805), and 1:1000 Hoescht in PBS, were incubated for 1 h at room temperature. Sections were then washed 3 × 5 min with PBS and mounted with Mowiol.

#### Cell colocalization for differentiation and proliferation

YFP+ cells on and within 200 μm of the corpus callosum were analyzed for the expression of DCX (neurons), Ki67 (proliferation), GFAP (astrocytes, neural stem cells), and CC1 (oligodendrocytes) on Axiovert 200 at 20× magnification. Images of DCX were also taken on an Axio Observer Z1 spinning disk optical sectioning system. Anti-GFP was used to identify YFP+ cells when antigen retrieval was performed.

#### Microglia and infiltrating macrophages

The numbers of Iba+ cells were counted adjacent to the cathode, anode and cell injection site using an inverted Zeiss Observer Z1 microscope at 20× magnification. A 200 × 200 μm square was counted medial and lateral to the needle tracks at 100 μm from the surface of the brain from two sections per brain.

### Migration analysis

Brains were sectioned on the cryostat with a thickness of 20 μm. Every fifth section (100 μm) was counted. Analysis was performed on brains perfused 1–2 h after both 3- and 6-d stimulated and non-stimulated mice.

Using a 5× objective and the mosaic function of Axiovision Observer Z2, the interhemispheric midline (here called midline) was identified and transplanted cells were imaged for each brain section. The midline was used as the medial-lateral reference point and the rostral anterior commissure was used as the rostral-caudal reference point for each brain. The distance of the dorsal-lateral corner of the ventricle and electrode sites was measured with respect to the midline and rostral anterior commissure. Analysis was done in sections 100 μm apart and included all sections with YFP+ cells on the corpus callosum or in the cortex (denoting the injection site), for a total of 5–15 sections per brain.

#### Injection site

The injection site was identified by the YFP+ cells in the cortex. The medial-lateral position of the injection site was obtained for each brain by averaging the distance from the midline to the center of the cortical YFP+ cells (medial-lateral) in each section. The anterior-posterior position of the injection site was the average distance of YFP+ cortical cells from the anterior commissure. Only brains whose injection sites were rostral to the anterior commissure were considered for the migration analysis.

#### Cell spread analysis

Medial and lateral migration was defined as the distance between the injection site and the most medial and lateral YFP+ cell, respectively. The cells analyzed were on or adjacent to the corpus callosum (here called “on the corpus callosum”). To obtain the medial and lateral migration, first the distance between the closest and farthest YFP+ cell to the midline was taken per brain. Then to determine how far the cell migrated, we calculated the difference between the most medial cell or lateral cell and the injection site per brain. These distances were averaged across brains to give the medial and lateral migration in the stimulated and non-stimulated group.

Rostral and caudal migration was defined as the distance between the injection site and the most rostral and caudal sections with YFP+ cells, respectively. Only brains that had a spread of cells on the corpus callosum were considered for migration analysis.

#### Medial-lateral YFP+ cell pixel relative percentage analysis

Images were exported as TIF files and sections were changed to 8 bits and filtered (filter = moments) to process YFP+ cell pixel counts. The corpus callosum was outlined using an overlaid brightfield image of the tissue section and pixel counts were done on the corpus callosum. Pixel counts were generated for cells in the injection site: the nominal inner diameter of the Hamilton needle was 133 μm, thus to account for this distance we counted pixels at 50 μm on either side of the brain’s average injection site as well as medial (cathodal) and lateral (anodal) from the injection site. For consistency, the section with the most medial cell was analyzed for each mouse brain.

#### Rostral-caudal and medial-lateral cell spread graphs

Each brain was plotted with the most medial and lateral cell per section, the injection sites, lateral dorsal corner of the lateral ventricle and the cathode and anode implant locations plotted with respect to the midline (*x*-axis, 0) and rostral anterior commissure (*y*-axis, 0). Cathode and anode electrode implant sites seen in brightfield or DAPI-stained brains, were included on the plots.

### Endogenous voltage measurements

To measure the electrical potential difference on the corpus callosum, mice were sacrificed and the brain was removed and placed in aCSF (room temperature). The olfactory bulb was removed and the brain was sectioned with one cut 2 mm caudal to the anterior frontal lobe (at the injection site and electrode site) and 2 mm caudal, generating two 2-mm-thick coronal sections per brain. Voltage measurements were performed in brain sections from mice that did not have electrode implants or cell transplants. Each measurement was performed with freshly cut insulated wire leads for a cleaner platinum electrode surface and to reduce possible biases based on the different platinum wire electrode surface areas for each cut. Four platinum wire two-lead insulated electrodes were used, and each measurement was taken with a different electrode or a freshly cut wire lead.

The measuring electrode was placed into a stereotactic holder and measurements were recorded on the microvoltmeter (QA350 Microcvolt DC Volt Meter, QuantAsylum) using application version 1.702 to perform text logging on slow DC mode. Recordings were performed until they stabilized which was defined as when the SD of the measurement was <10 μV after at least 100 data points. The voltage was measured in regular aCSF beside the brain slice to determine the baseline potential for the electrodes. The medial electrode lead was then placed 0.7 mm lateral from midline to mimic the stimulating electrode position *in vivo* and lowered 200–600 μm deep to ensure both tips were inserted into the corpus callosum. Measurements were taken between the two electrodes. Following this, the electrode was removed and measured once more in aCSF.

We measured the voltage between the medial electrode lead (positive input) with reference to the lateral electrode lead (negative input) on one hemisphere of the brain. To consider systemic variations, we then measured the voltage of the lateral electrode lead (positive input) with reference to the medial electrode lead (negative input) in the other hemisphere of the brain. We also measured the voltage of each lead (positive input) with respect to another reference insulated platinum wire electrode lead (negative input) that was ∼6 cm away in saline. The differences between the two electrode leads within the brain tissue seemed to be consistent with the measured voltages with respect to the reference electrode in saline. Therefore, the voltage difference on the corpus callosum was calculated by the difference between the two electrode leads in the brain tissue.

The recordings were processed using 1 min of recordings at the end of the reading and averages were calculated and data points >1 SD from the average were removed. The electric potential difference across the corpus callosum was taken as the measured value less the measured aCSF value and was averaged over each measurement per brain.

Although each measurement was taken with a different electrode as the positive input and negative input, the electric potential difference was adjusted to be the lateral electrode (positive input) and the medial value (negative input). Therefore, measurements taken with the medial electrode (negative input) and the lateral electrode (positive input) were multiplied by “–1” to account for the change in electrode positive and negative inputs.

### Experimental design and statistical analysis

Values are represented as mean ± standard error of the mean unless otherwise stated and further description of variables can be found in the results of the respective figures. Statistical analysis between the stimulated and non-stimulated groups was performed by an unpaired *t* test for the 3-d paradigm. An unpaired *t* test was used to analyze the pooled medial (stimulated and non-stimulated) versus pooled lateral (stimulated and non-stimulated) 3-d paradigm.

A multiple comparisons one-way ANOVA test with Tukey’s *post hoc* corrections was used in the 3-d stimulation paradigm to compare stimulated and non-stimulated brains; the percentage of cells on the medial-lateral side of the injection site; the number of Iba1+ around the injection site and electrode sites; and migration distance in the rostral-caudal directions. The same test was used in the 6-d paradigm to compare stimulated and non-stimulated brains for migration distances in medial-lateral and rostral-caudal directions.

A one-tailed *t* test was used to determine whether a potential difference existed on the corpus callosum.

**Figure 2.**
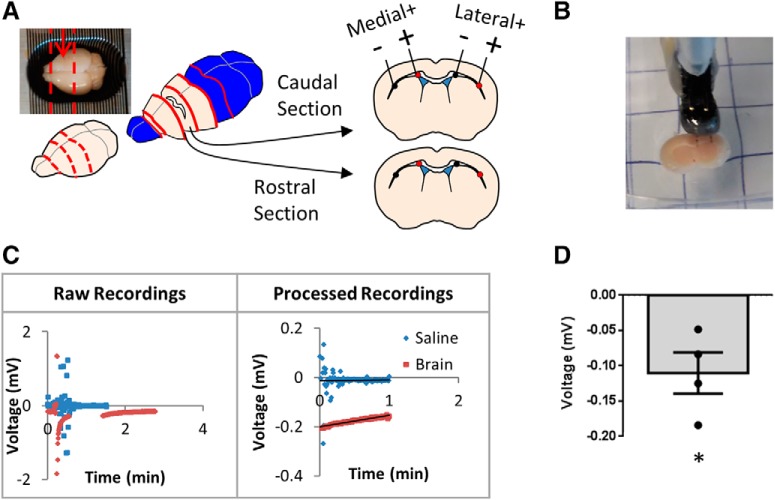
Endogenous voltage measurements. ***A***, Brain schematic depicting location of voltage measurements. Cuts are in dashed red lines and the arrow depicts rostral-caudal location of cell transplant and electrode implant. The red dot in the coronal section is the positive measurement location and the black dot is the negative. ***B***, Set-up of measurement on the corpus callosum. ***C***, Sample measurements of the medial and lateral side of a caudal section. ***D***, Voltage difference between the lateral and medial locations on the corpus callosum (*n* = 4 brains, 16 measurements). Each point in the graph represents an average in a mouse brain. Data represented as mean of all mouse brains ± SEM. One-sample *t* test was used and the voltage value was significantly different from zero, *p = 0.032^e^.

A multiple comparisons one-way ANOVA test with Sidak’s *post hoc* corrections was used to compare migration distance differences between 3- and 6-d stimulation paradigms in their respective stimulated and non-stimulated medial, lateral, rostral, or caudal migration direction. Statistical table for the analyses performed is provided in Table 1. Each line in the table refers to analyses performed throughout the text as indicated by superscripts lowercase letters.

## Results

### Electrical stimulation over 3 d increases migration toward the cathode in stimulated versus non-stimulated brains

We tested the unidirectional migration response to electrical stimulation in either the medial or lateral direction in stimulated and non-stimulated brains. As shown in Figure 1*D*, the medial migration from the injection site is greater in stimulated (491 ± 22 μm) versus implanted non-stimulated brains (404 ± 36 μm). The lateral migration distance was not significantly different between stimulated and non-stimulated groups (968 ± 67 vs 888 ± 77 μm, respectively; Fig. 1*E*). Interestingly, there seemed to be an innate bias to lateral migration. To investigate this phenomenon, we pooled the medial non-stimulated and stimulated brains and compared them to the pooled lateral non-stimulated and stimulated brains. There was significantly further lateral migration compared to medial (932 ± 50 vs 451 ± 22 μm, lateral and medial, respectively, unpaired *t* test, *p* < 0.0001)^c^. We also quantified the relative percentage of cells on either side of the injection site and found there were more cells on the lateral side of the injection site in both stimulated and non-stimulated brains (Fig. 1*F*). This suggests there may be a default lateral migratory path for these transplanted NPCs on the corpus callosum.

### Endogenous electrical signals are present in the corpus callosum

Having found that transplanted cells migrated laterally, irrespective of the presence of EF application, and considering previous work demonstrating that endogenous EFs exist on the RMS that correlate with endogenous neuroblast migration (Cao et al., 2013), we asked whether endogenous EFs were present in the corpus callosum that could potentially underlie lateral cell migration. As seen in Figure 2*A*,*B*, the medial and lateral electrodes served as the measuring and ground leads. Potentials were recorded and processed both in saline and in the brain (Fig. 2*C*). Potentials stabilized after an average of 3.6 ± 0.4 min after start of recording. The magnitude of the difference between slopes of the brain and their respective saline recordings was 0.02 ± 0.007 mV/mm. The recorded potentials were averaged over 1 min of stable recording and an average reading was generated per mouse brain (–0.11 ± 0.03 mV) which was significantly different from zero (Fig. 2*D*). Thus, these findings reveal that an endogenous EF exists along the medial to lateral corpus callosum which correlates with the robust lateral NPC migration of transplanted NPCs *in vivo*.

### Electrical stimulation does not change the differentiation profile of transplanted YFP+ cells

**Figure 3.**
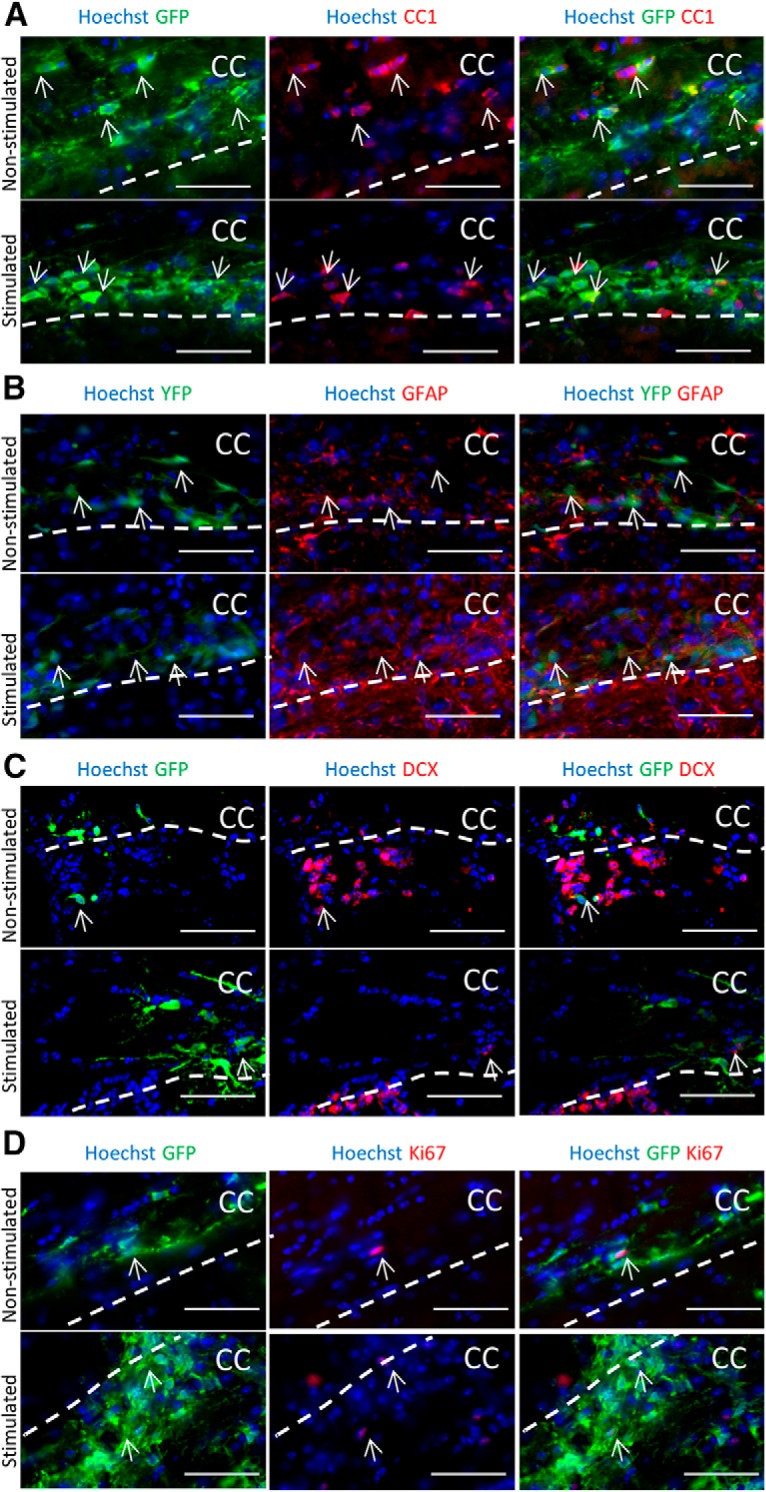
Three-day stimulation paradigm does not change the differentiation profile and proliferation of the transplanted cells. Images of YFP+ Hoescht+ cells colocalized with (***A***) CC1+ for oligodendrocytes (***B***) GFAP+ for astrocytes and neural stem cells, (***C***) DCX+ for neuroblasts, (***D***) Ki67+ for proliferating cells. Arrows depict colocalized cells. Dotted line approximates the margins of the corpus callosum (CC). Scale bar = 50 μm.

Previous studies have demonstrated that undifferentiated NPCs, but not differentiated cells, undergo galvanotaxis *in vitro* (Babona-Pilipos et al., 2011). Further, electrical stimulation *in vitro* does not change the differentiation profile of NPCs (Babona-Pilipos et al., 2011). Here, we asked whether stimulation changed the differentiation profile of the transplanted YFP+ cells on the corpus callosum *in vivo*. Mice were perfused 1–2 h after the last stimulation of the 3-d paradigm and brains were removed, sectioned, and analyzed. Sections were stained with markers for oligodendrocytes (CC1), immature neurons (DCX), and astrocytes and neural stem cells (GFAP). The numbers of colocalized YFP+ cells (labeled with anti-GFP; GFP+) was assessed. We observed no difference in the number of oligodendrocytes in stimulated and non-stimulated brains (CC1+/GFP+, 16 ± 5% and 14 ± 6%, non-stimulated and stimulated, respectively, four to six sections per brain, *n* = 3 brains per group, unpaired *t* test, equal variance, *p* = 0.8^f^; Fig. 3*A*). Expression of GFAP+/YFP+ cells on the corpus callosum was similar in both stimulated and non-stimulated brains (Fig. 3*B*). We found only rare instances of neurons (DCX+/GFP+) which were invariably found in the neurogenic, periventricular region of stimulated and non-stimulated brains (Fig. 3*C*). Finally, a small fraction of transplanted cells expressed the proliferation marker Ki67 (Ki67+/GFP+) and was not significantly different between stimulated and non-stimulated brains (4.0 ± 1.5% and 4.3 ± 0.7%, stimulated and non-stimulated, four to six sections per brain, *n* = 3 brains per group, unpaired *t* test, equal variance, *p* = 0.9^g^; Fig. 3*D*). Together, these findings indicate that stimulation did not promote NPC differentiation or proliferation following transplantation.

### Electrical stimulation does not increase Iba1+ microglia and macrophage cell number

**Figure 4.**
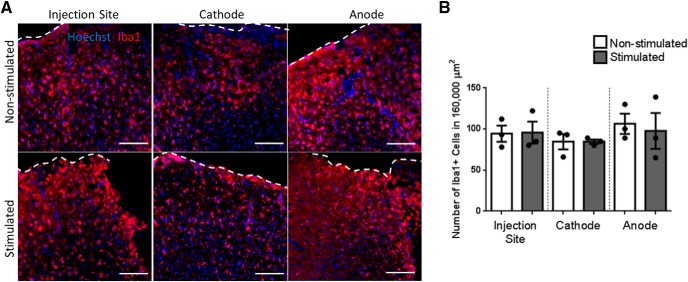
Three-day stimulation paradigm does not change Iba1+ cell number around implants. ***A***, Example Iba1+ response by non-stimulated and stimulated injection site, cathode and anode leads. ***B***, Number of Iba1+ cells around the electrode leads and injection site in non-stimulated (*n* = 3) and stimulated brains (*n* = 3). Each point in the graph represents the number of Iba1+ cells around the injection, or electrode lead in one mouse brain, plotted with mean ± SEM. A multiple comparisons one-way ANOVA test with Tukey’s *post hoc* corrections was used and there was no significant difference between the groups (*p* = 0.8)^h^. Dotted line depicts the surface of the cortex. Scale bars = 100 μm.

An important consideration is whether the inflammatory response of the brain is altered by EF application. The number of Iba1+ cells around the injection site, cathode, and anode was assessed. Iba1 is a marker of microglia and infiltrating macrophages, indicators of inflammation. As seen in Figure 4*A*,*B*, the number of Iba1+ cells was not significantly different between stimulated and non-stimulated brains, at all sites examined (injection site: 96 ± 13 vs 94 ± 10 cells; cathode: 85 ± 3 vs 85 ± 9 cells; anode: 98 ± 22 vs 106 ± 12 cells, stimulated vs non-stimulated, respectively). Hence, stimulation was not leading to an increased inflammatory response *in vivo*.

### Electrical stimulation and rostral-caudal migration

**Figure 5.**
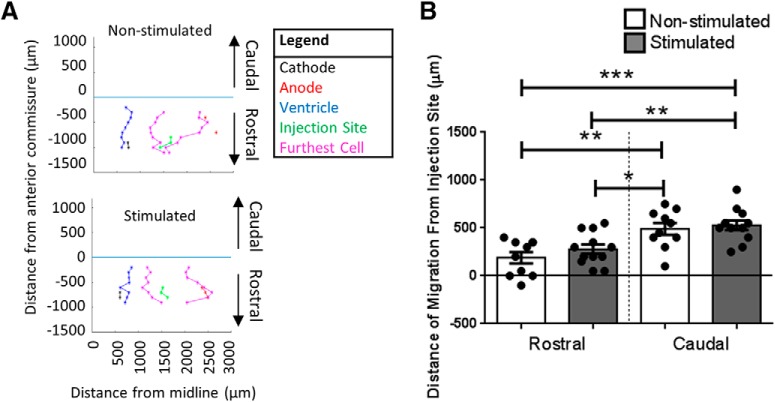
Three-day stimulation paradigm cell spread in the rostral and caudal directions. ***A***, Example plots of an implanted non-stimulated and stimulated brain. ***B***, Migration from the injection site in the rostral, caudal and lateral direction in implanted non-stimulated (*n* = 10) and stimulated (*n* = 12) brains. Each point in the graph represents the farthest rostral or caudal migrating cell in a mouse brain, plotted with mean ± SEM. A multiple comparisons one-way ANOVA test with Tukey’s *post hoc* corrections was used, and there were significant difference in migration directions (*p = 0.046, **p < 0.01, ***p = 0.0005)^i^.

Based on *in vitro* experiments, we made the strong prediction that NPCs would migrate cathodally in the presence of an EF *in vivo*. However, *in vivo*, endogenous environmental cues are present in addition to the applied electrical stimulation and the transplanted cells can migrate in three dimensions, compared to only two dimensions *in vitro*. Hence, while we did observe increased cathodal migration, we also noted considerable spread of transplanted cells away from the injection site in both the stimulated and non-stimulated brains, in the lateral direction (Fig. 1*C*). To gain a better understanding of the overall cell spread, we mapped the location of the cells in the rostral-caudal direction throughout the brain (Fig. 5*A*). The locations of the cells closest to the midline and farthest from the midline were plotted, as well as the location of the ventricle at their respective rostral-caudal position from the injection site for reference. The placement of the electrodes was also noted when visible in the tissue. There was no significant difference between stimulated and non-stimulated groups for the rostral and caudal direction (caudal: 529 ± 50 and 490 ± 63 μm, rostral: 279 ± 49 and 190 ± 59 μm, stimulated vs non-stimulated groups, respectively; Fig. 5*B*). Interestingly, regardless of stimulation, the cells moved further caudal compared to rostral. Taken together, the preference for lateral and caudal migration, regardless of exogenous EF application, and the presence of an endogenous EF along the corpus callosum in the medial-lateral direction highlights the presence of endogenous environmental cues regulating NPC migration in the brain.

### Electrical stimulation over 6 d could affect default migratory paths

**Figure 6.**
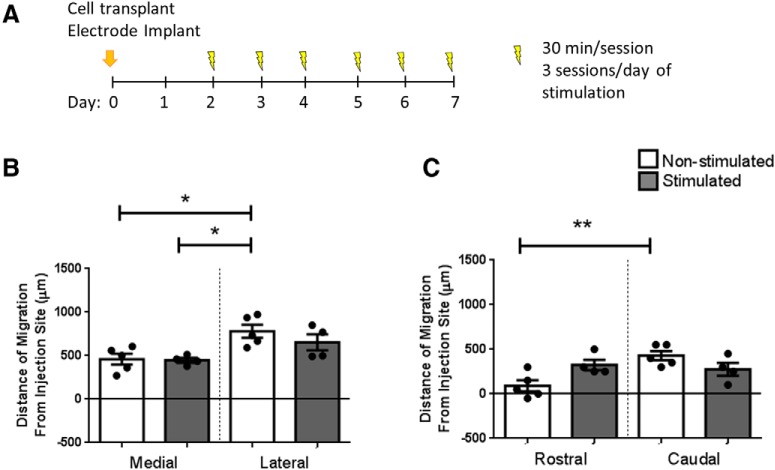
Six-day stimulation paradigm could affect default migratory paths. ***A***, Electrical stimulation paradigm for 6-d stimulation. Medial and lateral (***B***) and rostral and caudal (***C***) migrations were analyzed in implanted non-stimulated (*n* = 5) and stimulated brains (*n* = 4). Each point on the graph represents a different farthest migrating cell in a mouse brain, mean ± SEM. A multiple comparisons one-way ANOVA test with Tukey’s *post hoc* corrections was used in the medial-lateral migration direction and the rostral-caudal migration direction which showed differences in migration distances after stimulation (*p < 0.05, **p = 0.0048)^j,k^.

In a final series of experiments, we asked whether we could overcome the lateral migration *in vivo* by increasing the number of stimulation days (Fig. 6*A*). Interestingly, when stimulation duration was doubled, there was no significant difference in migration toward the midline (460 ± 63 vs 448 ± 28 μm, non-stimulated vs stimulated) or away from the midline (781 ± 75 vs 653 ± 92 μm, lateral migration non-stimulated vs stimulated; Fig. 6*B*). Of note, unlike in the 3-d paradigm this longer stimulation paradigm did not result in a significant difference between lateral migration distance and medial migration distance. Similarly, after stimulation, caudal migration compared to rostral migration distance was not significantly different (Fig. 6*C*). Interestingly, 6 d of stimulation compared to 3 d of stimulation resulted in a significant decrease in the lateral migration (multiple comparisons one-way ANOVA, Sidak’s *post hoc* corrections, *p* = 0.013)^l^. Thus, the default preference for lateral and caudal migration can be disrupted by prolonged stimulation.

## Discussion

A number of studies have investigated NPC migration in the presence of EFs, termed galvanotaxis (galvanotaxis) *in vitro* (Babona-Pilipos et al., 2011, 2015, 2018). In this study, we investigated the effects of biphasic charge-balanced electrical stimulation for galvanotaxis of transplanted NPCs *in vivo* in the mouse brain. We found that transplanted NPCs had a propensity to migrate laterally along the corpus callosum under baseline conditions and established that endogenous electric potential differences exist along the corpus callosum (more negative laterally compared to medially). This endogenous EF is consistent with the default migration pathway of transplanted NPCs revealing that the corpus callosum is an endogenous migratory pathways that utilizes EFs as a guidance cue in the brain (Cao et al., 2013; Feng et al., 2017). Further, we determined that an applied EF (3-d paradigm) was able to enhance the cathodal distance of cell migration on the corpus callosum, while longer stimulation (6-d paradigm) reduced the caudal and lateral NPC migration. Together, these findings support that EFs play a role in NPC migration and are important considerations for neural repair.

The small, but significant, stimulation effect on NPC migration was based on parameters that were optimized for *in vitro* application (Babona-Pilipos et al., 2011, 2015). To further enhance NPC migration in a charge-balanced EF, optimization of the stimulation paradigm, in terms of duration, pulse widths, frequency, and amplitudes will be needed. Challenges around optimizing the migration parameters include the heterogeneity of the brain parenchyma resulting in regionally distinct EFs (Kuncel and Grill, 2004; Brocker and Grill, 2013). We predicted that the cells would migrate toward the cathode based on *in vitro* work and consistent with the EF lines of an applied field between two parallel wires (Fig. 1*B*); however, these depicted EF lines do not take into account the different conductivities of the tissue (extracellular matrix, white matter and gray matter). This heterogeneity could affect the applied EF lines and cell migration directions. An important next step will include the modeling EF distribution to aid in the optimization of stimulation parameters to promote migration.

In this study, we found that the transplanted cells that survived and migrated *in vivo* were primarily found along the borders (dorsal and ventral) of the corpus callosum and were not within the gray matter. This could be due to the different conductivities of the gray and white matter creating a potential difference at the interface of the two tissues and directing cells to these borders (Brocker and Grill, 2013). Further, cell migration was more extensive in the corpus callosum compared to the cortex, where cells survived, but tended to be concentrated at the site of injection. This observation highlights the importance of the microenvironment which includes endogenous EFs, cytoarchitecture, and cellular components, which have all been shown to influence galvanotaxis even to the point of causing the cells to migrate in opposite directions (Huang et al., 2016; Iwasa et al., 2017, 2018). As such, the location of the electrodes in the cortex may not have been optimal for the cells to perceive and migrate in the EF. Further, the cells migrating along the margins of the corpus callosum toward the cathode placed medially near the ventricle may have been exposed to repulsive cues known to be present within the lateral ventricles (Kaneko et al., 2017), thereby limiting the distance the cells migrated in the 3- and 6-d paradigms. Of note, even in the absence of stimulation, the 3- and 6-d paradigm revealed a significant difference between the caudal/rostral migrations and between the medial/lateral migrations.

Endogenous EFs within the brain, and generated in response to injury can influence cell migration. Indeed, we established the presence of endogenous EFs in the medial-lateral axis on the corpus callosum and expect that endogenous EFs will be present in other regions including along the rostral-caudal axis. Measuring this EF and investigating the importance of native bioelectric cues is challenging and important. We used electrodes of a larger diameter of 127 μm to reduce the possibility of measuring a single cell’s membrane potential. Interesting next steps might involve disrupting the EF by applying an EF in the opposite direction in the rostral-caudal axis. We could collapse the native EF with blockers, disrupt the EF by applying an EF in the opposite direction in the rostral-caudal direction on the corpus callosum and perform computational modeling on our cell migration predictions. Computational modeling of the effects of EFs on cells, based on parameters in this work and others (Cao et al., 2013; Thrivikraman et al., 2018), is an important future direction when considering exogenous EF application as a therapeutic strategy.

The lineage and differentiation state of cells can also modify their response to EFs (Patel and Poo, 1982; Babona-Pilipos et al., 2011; Baer et al., 2015; Li et al., 2015). With respect to NPCs, we have shown that undifferentiated NPCs migrate in the presence of EFs but not their differentiated progeny (Babona-Pilipos et al., 2011). Since we did not observe robust, cathodal NPC migration *in vivo*, we examined the differentiation state of the transplanted cells and observed that only a small subpopulation of cells present on the corpus callosum after 3 d were mature cell types. The relative percentage of differentiated cells did not appear different between stimulated and non-stimulated brains, which is consistent with *in vitro* studies showing that EF application does not promote cell differentiation (Babona-Pilipos et al., 2011, 2015). We cannot rule out the possibility that the 6-d stimulation paradigm resulted in increased cell differentiation or changes in cell survival, and this accounted for the loss of cathodal migration between stimulated and non-stimulated brains.

This article demonstrates the potential of using charge-balanced biphasic electrical stimulation to direct cell migration. We highlight that the microenvironment, including endogenous EFs, are important cues to consider when applying electrical stimulation to direct cell migration. Measuring other electric potential differences in the brain including in the rostral-caudal direction on the corpus callosum could provide clues to other migratory cell paths in the brain both during development and adulthood. Utilizing a combination of these default migratory paths and applied electrical stimulation to direct cells to damaged tissue could be a promising approach for tissue repair.

**Table 1.** Statistical table

	Data structure	Type of test	Confidence interval	
a	Normal	Unpaired *t* test	2 to 172	
b	Normal	Unpaired *t* test	–132 to 293	
c	Normal	Unpaired *t* test	–591 to –370	
d	Normal	Multiple comparisons one-way ANOVA test with Tukey’s *post hoc* corrections	Non-stim medial versus non-stim lateral	–76 to –8
			Non-stim medial versus stim medial	–34 to 32
			Non-stim medial versus stim lateral	–74 to –9
			Non-stim lateral versus stim medial	9 to 74
			Non-stim lateral versus stim lateral	–32 to 34
			Stim medial versus stim lateral	–71 to –9
e	Normal	One sample *t* test	–0.20 to –0.02	
f	Normal	Unpaired *t* test	–23 to 19	
g	Normal	Unpaired *t* test	–5 to 4	
h	Normal	Multiple comparisons one-way ANOVA test with Tukey’s *post hoc* corrections	Non-stim Injection versus stim injection	–62 to 60
			Non-stim injection versus non-stim cathode	–51 to 71
			Non-stim injection versus stim cathode	–51 to 71
			Non-stim injection versus non-stim anode	–73 to 49
			Non-stim injection versus stim anode	–64 to 58
			Stim injection versus non-stim cathode	–50 to 72
			Stim injection versus stim cathode	–50 to 72
			Stim injection versus non-stim anode	–72 to 50
			Stim injection versus stim anode	–63 to 59
			Non-stim cathode versus stim cathode	–61 to 61
			Non-stim cathode versus non-stim anode	–83 to 39
			Non-stim cathode versus stim anode	–74 to 48
			Stim cathode versus non-stim anode	–83 to 39
			Stim cathode versus stim anode	–74 to 48
			Non-stim anode versus stim anode	–52 to 70
i	Normal	Multiple comparisons one-way ANOVA test with Tukey’s *post hoc* corrections	Non-stim rostral versus stim rostral	–298 to 119
			Non-stim rostral versus non-stim caudal	–518 to –82
			Non-stim rostral versus stim caudal	–548 to –131
			Stim rostral versus non-stim caudal	–419 to –3
			Stim rostral versus stim caudal	–449 to –51
			Non-stim caudal versus stim caudal	–248 to 169
j	Normal	Multiple comparisons one-way ANOVA test with Tukey’s *post hoc* corrections	Non-stim lateral versus stim lateral	–159 to 415
			Non-stim lateral versus non-stim medial	51 to 592
			Non-stim lateral versus stim medial	47 to 620
			Stim lateral versus non-stim medial	–93 to 480
			Stim lateral versus stim medial	–97 to 507
			Non-stim medial versus stim medial	–275 to 299
k	Normal	Multiple comparisons one-way ANOVA test with Tukey’s *post hoc* corrections	Non-stim rostral versus stim rostral	–488 to 18
			Non-stim rostral versus non-stim caudal	–578 to –102
			Non-stim rostral versus stim caudal	–438 to 68
			Stim rostral versus non-stim caudal	–358 to 148
			Stim rostral versus stim caudal	–216 to 316
			Non-stim caudal versus stim caudal	–98 to 408
l	Normal	Multiple comparisons one-way ANOVA with Sidak’s *post hoc* corrections	Non-stim rostral versus non-stim rostral 6 d	–161 to 361
			Stim rostral versus stim rostral 6 d	–321 to 229
			Non-stim caudal versus non-stim caudal 6 d	–201 to 321
			Stim caudal versus stim caudal 6 d	–21 to 529
			Non-stim lateral versus non-stim lateral 6 d	–154 to 368
			Stim lateral versus stim lateral 6 d	40 to 590
			Non-stim medial versus non-stim medial 6 d	–317 to 205
			Stim medial versus stim medial 6 d	–232 to 318

## Synthesis

Reviewing Editor: Chay Kuo, Duke University Decisions are customarily a result of the Reviewing Editor and the peer reviewers coming together and discussing their recommendations until a consensus is reached. When revisions are invited, a fact-based synthesis statement explaining their decision and outlining what is needed to prepare a revision will be listed below. The following reviewer(s) agreed to reveal their identity: Min Zhao. Note: If this manuscript was transferred from JNeurosci and a decision was made to accept the manuscript without peer review, a brief statement to this effect will instead be what is listed below.

Reviewer 1

The paper is improved and can be published essentially as-is. The one thing I would suggest (not requiring external review) is to improve the utility of Fig. 1A,B by showing how the field lines in B look on the actual tissue of the right panel of 1A. Right now, panel B is a simple picture of field lines from any physics text, which is fine, but that's not the critical part readers will be wanting to see: what we want to see is how the field lines overlay onto the anatomy of the brain relative to the electrodes and the migration paths of the cells. If possible, I would like to see a single bigger panel in 1B showing the brain, the electrodes, field lines, and where the cells are going, so that it's more obvious how the field relates to the migration paths of the cell (the biology being studied). I suggest the authors try, and if the result does not look to the editor like it's an improvement, then just publish as-is.

Reviewer 2

1. In Fig1D-F, although the P values are less than 0.05, the percentage of cell medial migration was low. It is also showed in Fig 5 and 6. Does it mean the electric stimulation work but with low efficiency? The authors perhaps can test different stimulating strength or suggest this issue and a requirement of future experiments.

2. The endogenous electric field measurement from Fig2 was done in saline and ex vivo brain section, which is different from the endogenous electric field potential in hemisphere, which should be doable. The measurements could be complicated by cell membrane potentials. How such complication can be ruled out?
