# Supplementary figures and images for: Charge-Balanced Electrical Stimulation Can Modulate Neural Precursor Cell Migration in the Presence of Endogenous Electric Fields in Mouse Brains
Source: eNeuro. 2019 Dec 13;6(6):ENEURO.0382-19.2019. doi: 10.1523/ENEURO.0382-19.2019 (PMC6978916; doi:10.1523/ENEURO.0382-19.2019)

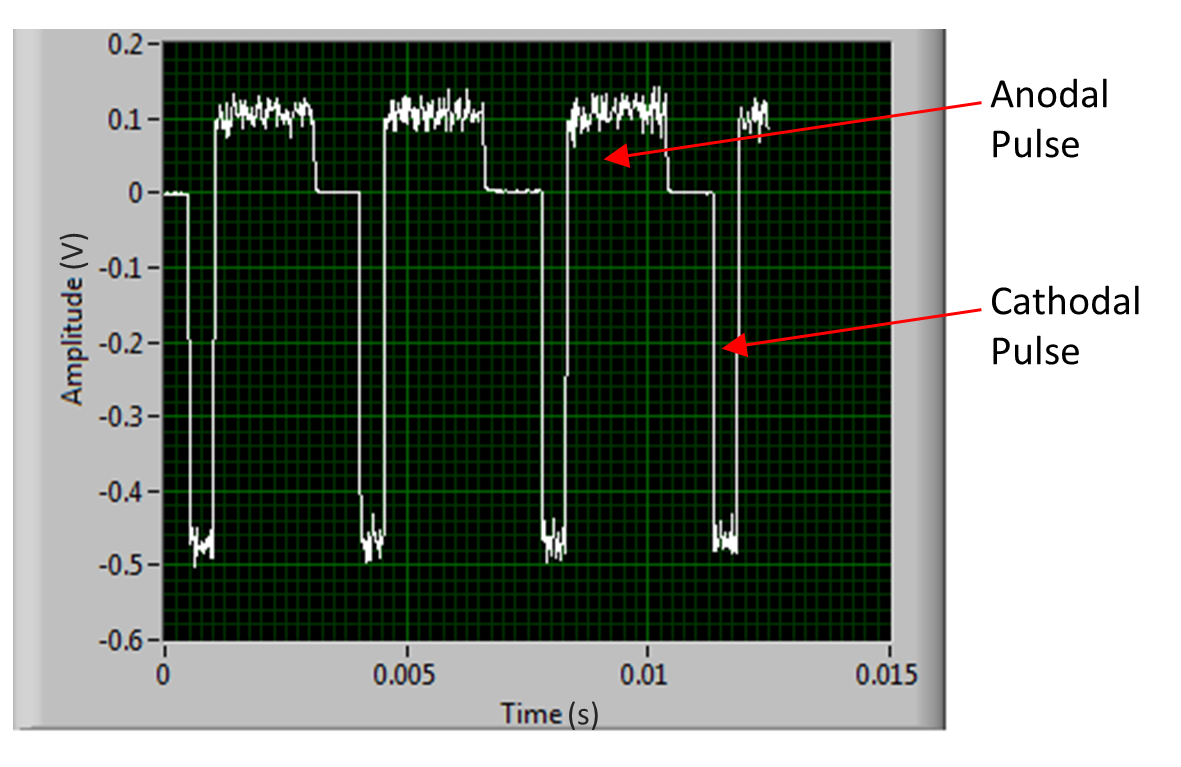

Supplement: Extended Data Figure 1-1 — Measured voltage waveform across the implanted electrode. Biphasic monopolar waveform consisting of a cathodal pulse with four times the amplitude of the anodal pulse. Pulse width of the anodal pulse is four times the duration in order to have a charge-balanced waveform. Download Figure 1-1, TIF file. [file sup_enu-eN-CFN-0382-19-s02.tif]
